# Patterns of healthcare use among children with immigrant and non-immigrant backgrounds in 2019 and 2020: evidence from the CRIAS cohort study in the metropolitan area of Lisbon, Portugal

**DOI:** 10.1186/s12889-023-17402-z

**Published:** 2023-12-18

**Authors:** Zélia Muggli, Thierry E. Mertens, Regina Amado, Dora Vaz, Helena Loureiro, Maria Rosário O. Martins

**Affiliations:** 1 Global Health and Tropical Medicine, Institute of Hygiene and Tropical Medicine, NOVA University Lisbon, Lisbon, Portugal; 2 Amadora Primary Care Health Centres Group, Regional Health Administration of Lisbon and Tagus Valley, Amadora, Portugal; 3 Department of Paediatrics, Hospital Prof. Dr. Fernando Fonseca, Amadora, Portugal

**Keywords:** Immigrant children, Healthcare use inequalities, Strengthening primary healthcare

## Abstract

**Background:**

International migrant families may face various barriers in the access and use of health services. Evidence on immigrant children’s health care or prevention facilities’ utilisation patterns is scarce in Portugal. Therefore, the objectives of this study were to compare health services use between immigrant and non-immigrant children in the Metropolitan Area of Lisbon in 2019–2020 with the aim of informing public policies towards equitable access to, and use of health services.

**Methods:**

The CRIAS (Health Trajectories of Immigrant Children) prospective cohort study enrolled 420 children (51.6% immigrant) born in 2015 and attending primary health care (PHC) services in 2019. We compared primary health care facilities and hospital paediatric emergency department (ED) utilisation patterns in the public National Health Service, together with reported private practitioners use, between immigrant and non-immigrant children in 2019 and 2020. The Pearson chi-squared test, Fisher-Freeman-Halton Exact test, two-proportion z-test and Mann‒Whitney U test were used to examine the differences between the two groups.

**Results:**

In 2019, no significant differences in PHC consultations attendance between the two groups were observed. However, first-generation immigrant children (children residing in Portugal born in a non-European Union country) accessed fewer routine health assessments compared to non-immigrant children (63.4% vs. 79.2%). When children were acutely ill, 136 parents, of whom 55.9% were parents of non-immigrant children, reported not attending PHC as the first point of contact. Among those, nearly four times more non-immigrant children sought healthcare in the private sector than immigrant children (*p* < 0.001). Throughout 2019, immigrant children used ED more often than non-immigrant children (53.5% vs. 40.4%, *p* = 0.010), as their parents reported difficulties in accessing PHC. In 2020, during the COVID-19 pandemic, fewer immigrant children accessed PHC compared to non-immigrant children (70% vs. 80%, *p* = 0.018). Both non-immigrant and immigrant children reduced ED use by 2.5 times, with a higher decrease among immigrant children (46% vs. 34%). In both 2019 and 2020, over 80% of immigrant and non-immigrant children used ED for conditions classified as having low clinical priority.

**Conclusion:**

Beyond identifying health care use inequalities between immigrant and non-immigrant children, the study points to urgent needs for public policy and economic investments to strengthen PHC for all children rather than for some.

**Supplementary Information:**

The online version contains supplementary material available at 10.1186/s12889-023-17402-z.

## Background

Many extraordinary challenges face migrant populations. They leave their cultural roots and modes of existence to embark on often harsh travelling conditions and land in unknown destinations where they have to swiftly adapt. While attempting to settle, migrant families often end up living in precarious conditions that are likely to affect adult and child health outcomes [1]. Migration has therefore been recognised as a social determinant of health for at least a decade or more [2–4].

Political instability and climate change drive an increasingly diverse and complex flow of international migrants [5, 6]. In 2021, non-European Union (EU) citizens accounted for 5.3% of the total EU population [7]. In Portugal, the same year, the proportion of non-EU nationals made up 5.1% of the total population [8, 9]. In connection to Portugal’s colonial past, migrant populations (henceforth named immigrant populations) originate predominantly from Brazil (29.3%) and Portuguese-speaking African countries (13.6%). Recently, a more diverse influx comes from South Asian countries [9]. There is, therefore, a great deal of heterogeneity in experiences among immigrant populations in Portugal, as elsewhere in Europe. In principle, such heterogeneity in origins and experiences should encourage health policies and planning processes to guide health services adaptation to continue welcoming immigrant and non-immigrant children alike. Yet, current research in the EU, as well as in Portugal, indicates that public policies lag behind in adapting health services to foster equitable access and use of healthcare by immigrant populations [10–14], including children [15–24].

Many barriers contribute to inequalities in sound and regular health prevention and care for children. They include language, length of stay, financial, legal, socio-cultural or administrative barriers. Furthermore, immigrant families from various origins may have different health needs and expectations, while sometimes facing discrimination based on country of origin, documentation status, ethnicity or religion in accessing healthcare [25–29]. Racism towards racialized immigrants in accessing and receiving healthcare has been observed at interpersonal and structural levels and constitutes a barrier to equity in healthcare. Racism has been associated with delay in seeking healthcare, lack of trust, feelings of being ignored and with differential medical treatment [30–32].

Since 1976, the Portuguese Constitution declared healthcare provision to be universal and needs-based through the tax funded public National Health Service (“Serviço Nacional de Saúde”, SNS), much alike the so-called “Beveridge system” in the United Kingdom. In addition, public and private subsystems cover particular professional sectors together with private voluntary health insurance, granting access to the private healthcare sector alongside the SNS [33], thereby introducing differential, and potentially unequal, patterns of health care utilisation. For children, the SNS is, in principle, universal and free up to the age of 18. Public healthcare provision includes specialist and hospital care as well as primary health care, covering preventive measures, e.g., vaccination and routine child health assessments. These assessments, of great importance to harmonious child development, involve monitoring growth and development from the child’s first week of life to the 18th birthday, together with age-related advice on various health topics. They also provide an opportunity to establish bridges with sectors other than health for the well-being of the child. Of recent introduction in Portugal, the SNS24 telephone helpline serves as a “gateway” to the SNS through “triage” and, when possible, follow-up and referral services [34].

Although migrant child health research was identified as a priority in 2018 by the UCL-Lancet Commission and the World Health Organisation (WHO) European Regional Office [1, 35], studies in Europe on healthcare utilisation have been largely focussing on adult immigrant populations.

The objectives of this study were to describe reported children’s health care utilisation patterns by parents and to evaluate whether there were differences in primary healthcare and hospital emergency department use between immigrant and non-immigrant children in 2019 and 2020 in the CRIAS cohort study in the Metropolitan Area of Lisbon with the view to inform public policy to reduce eventual inequalities.

## Methods

### Study design and setting

This paper used cross-sectional data from the 1st and 2nd wave data sets of the CRIAS cohort study. Established in 2019 in Amadora Municipality (part of the Lisbon Metropolitan Area), the CRIAS cohort study uses a life-course approach to assess whether immigrant children present different health outcomes, including physical and psychomotor development, emotional and behavioural challenges and healthcare use patterns over time, when compared to non-immigrant children. In the study, an *immigrant child* was defined as a child residing in Portugal and born in a non-EU country (1st generation immigrant) or having at least one parent born in a non-EU country. The term *non-immigrant child* refers to a child born in Portugal to parents both born in Portugal.

In 2020, 13% of Amadora’s population was made of foreign nationals (977/km^2^) [9, 36]. Until December 2020, this municipality was served by 9 Primary Health Care Centres and one referral hospital – “Hospital Prof. Dr. Fernando Fonseca (HFF)” with a paediatric emergency department (ED). Children’s recruitment for the CRIAS cohort was carried out in the PHC centres. A detailed description of the CRIAS cohort study has been published elsewhere [37].

### Participants

Children born in 2015, age 4 at the start of the CRIAS study, with records of attending the health centre in the previous 2 years, were eligible to take part in the study. There were 1009 children fulfilling these criteria. Based on a previous study, 30% of users were immigrant children which corresponds to 302 children. To increase the power of comparisons overtime between immigrant and non-immigrant children, we aimed to have a proportion of 50% of each resulting in a total of 604 children eligible to participate. Initially planned from June 2019 to June 2020, recruitment for the study had to be interrupted in March 2020 due to the COVID-19 pandemic. During recruitment, 499 parents/caregivers were invited to enrol; a participation rate of 84% resulted in 420 children being included in the study, 217 (51.7%) of whom were immigrants.

### Data collection

Data on the family’s socioeconomic and demographic characteristics, migration history and child health were collected at the time of recruitment at age 4/5 through face-to-face interviews using the CRIAS baseline structured questionnaire. Open-ended questions were included to collect information about parents’ healthcare-seeking experiences for their child. Data regarding PHC and ED utilisation in 2019 and 2020 were retrieved from health centres and hospital electronic medical records, respectively. These two data sets and the baseline questionnaire were integrated using a key, accessible by only one investigator, linking the SNS user number with the child’s study ID.

### Outcomes related to health care use and other variables

The main outcomes of interest were the utilisation patterns of primary health care facilities and of the hospital paediatric emergency department. Primary health care (PHC) utilisation was defined for three levels: attendance to at least 1 consultation in 2019 and 2020 (yes/no), the number of consultations per year and participation in the yearly routine child health assessment at ages 4 and 5 (yes/no). Use of ED was defined by at least 1 visit in 2019 and 2020 (yes/no), number of visits per year and “frequent user” status expressed as parents taking their child to visit ED 4 or more times/year (yes/no).

For a deeper understanding of the use of ED, variables such as the origin of the patient on arrival at the ED, clinical priority classified by the Manchester Triage System (MTS) and inpatient admissions were also collected and evaluated. The MTS is a clinical risk management tool used to determine the target waiting time suitable to ensure the safety of the patient admitted to the ED. The system assigns a colour to the patient based on clinical priority [38]. Presenting conditions in the ED were classified by the hospital as per the International Classification of Diseases, Ninth Revision, Clinical Modification (ICD-9-CM) [39].

Data on the sociodemographic profile of children and their parents/caregivers were also collected and analysed. Assignment to a family doctor (yes/no) and private health insurance coverage beyond the SNS (yes/no) were also evaluated. Furthermore, we explored information on children’s attendance at PHC when acutely ill and the reasons given to visit the ED in those cases when the child had used the service in the 3 months prior to the interview.

### Statistical analysis

Data were disaggregated for migration status as “immigrant” or “non-immigrant” children, according to the respective study definitions. To assess the difference in the distribution of categorical variables between the two groups, we used the Pearson chi-squared test, and when assumptions were not met, the Fisher-Freeman-Halt exact test was carried out. The Mann–Whitney U test was used to compare quantitative variables. The two-proportion z-test was used to compare proportions of dichotomous variables of healthcare utilisation. Answers to the open-ended questions were systematically coded and categorised for quantitative content analysis.

Data analysis was conducted using the Software Package for the Social Sciences (SPSS®) version 27.0 (IBM Corp., Armonk, NY, USA). The significance level used was 5%.

## Results

### Characteristics of children and their parents/caregivers

The main countries of origin of immigrant parents/caregivers were Portuguese-speaking African countries (60%), followed by Brazil (13%). Among the 217 (51.7%) immigrant children, 41 (18.9%) were born in a non-EU country (defined as a first-generation immigrant in this paper), 75% originating from the community of Portuguese-speaking countries. Children from countries such as India, Nepal or Eritrea were also enrolled in the CRIAS cohort. Immigrant’s mother’s length of stay in Portugal had a median of 9 years (min. 0.1-max. 37), and further characteristics of children and families are summarised in supplemental Table 1. Immigrant children live significantly more often in very low-income (household income < 500€/month) families (18.5% vs. 6.7%, *p* < 0.001) with their parents/caregivers employed in poorly skilled occupations (35.2% vs. 10.0%, *p* < 0.001), when compared with non-immigrant children. Fewer immigrant children had an assigned family doctor (73.9% vs. 88.6%, *p* < 0.001), with only 37% of first-generation immigrant children having access to an assigned family doctor. Non-immigrant children benefited from health insurance schemes covering healthcare beyond SNS more frequently (51.5% vs. 29.3%, *p* < 0.001).

### Utilisation of primary health care facilities

In 2019, a cumulative total of 78% of immigrant children attended PHC for at least 1 consultation in comparison with a cumulative total of 73.8% of non-immigrant children (*p* = 0.312), with virtually no difference in the number of consultations between the two groups. In contrast, in 2020, more non-immigrant children (80.2% vs. 70.2%; *p* = 0.018) used PHC and had a higher number of consultations compared to immigrant children.

**Fig. 1:**
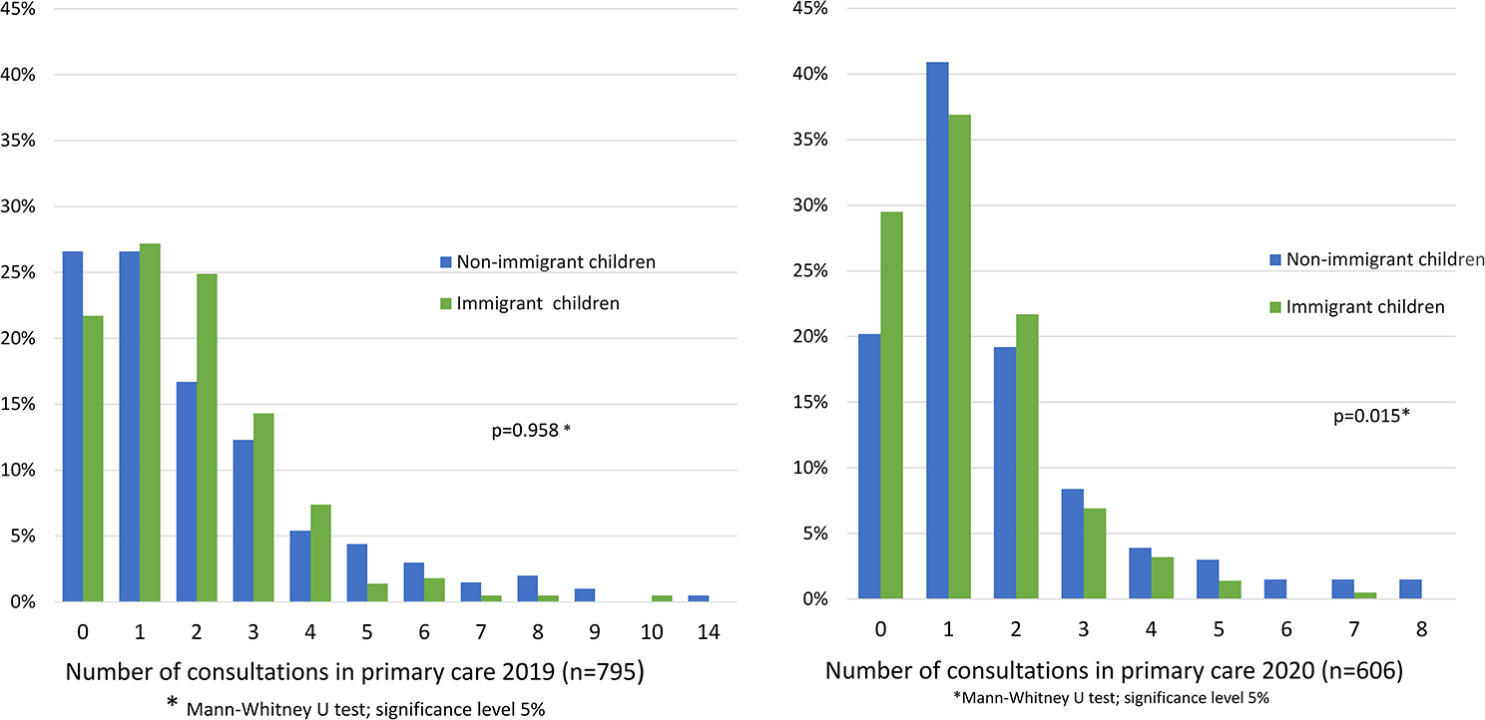
Number of consultations in primary care for immigrant and non-immigrant children in 2019 and 2020

The results are shown in Fig. 1.

Although there were no overall significant differences in attendance at routine health assessments at ages 4 and 5 between non-immigrant and immigrant children (supplemental Fig. 1), fewer 1st generation immigrant children (18,3% of all immigrant children) attended their assessments at age 4 (63.4% vs. 79.2%) and age 5 (24.4% vs. 31.5%) when compared with non-immigrant children. In the context of the COVID-19 pandemic, a dramatic reduction of circa 60% was observed in the total number of children who had the opportunity to participate in routine health assessments for age 5 (2020/21) compared with routine health assessments for age 4.

### Parents’ reported reasons for not seeking PHC when children are acutely ill

**Fig. 2:**
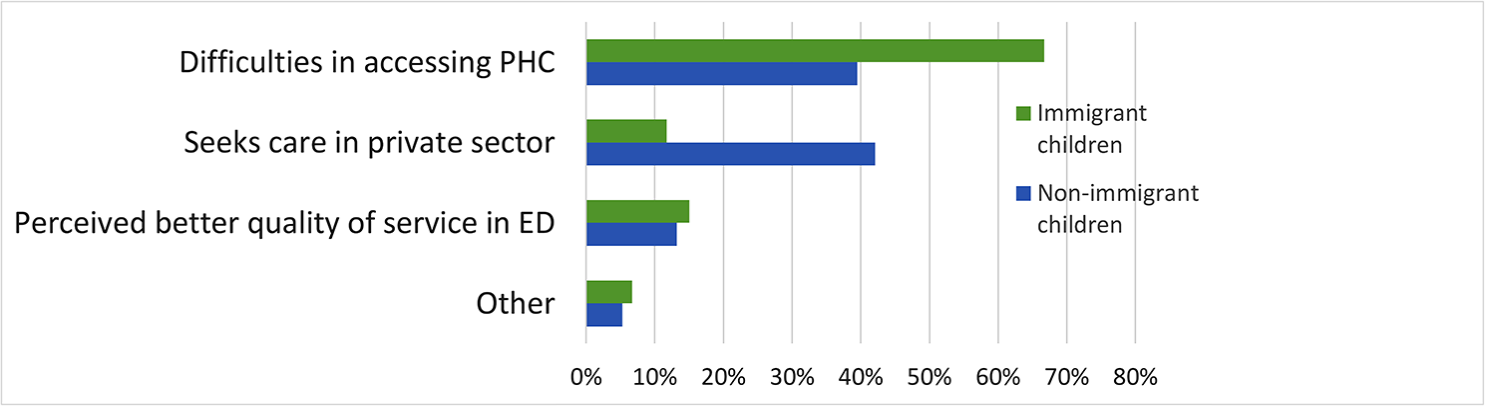
Reasons given by parents for not using primary care when child is ill (n = 136)

Parents of 136 children, of whom 55.9% were non-immigrant children, reported not attending PHC as the first point of contact when children are acutely ill. The main reasons given are shown in Fig. 2. Among parents of non-immigrant children, the main reason provided was seeking healthcare in private health facilities, reported nearly 4 times more than by parents of immigrant children. Difficulties in accessing PHC, including lack of availability of appointments on the day or impossibility to establish phone/email contact with the health centre or inconvenient opening times, were the main reasons given by parents of immigrant children.

### Utilisation of hospital emergency department

**Fig. 3:**
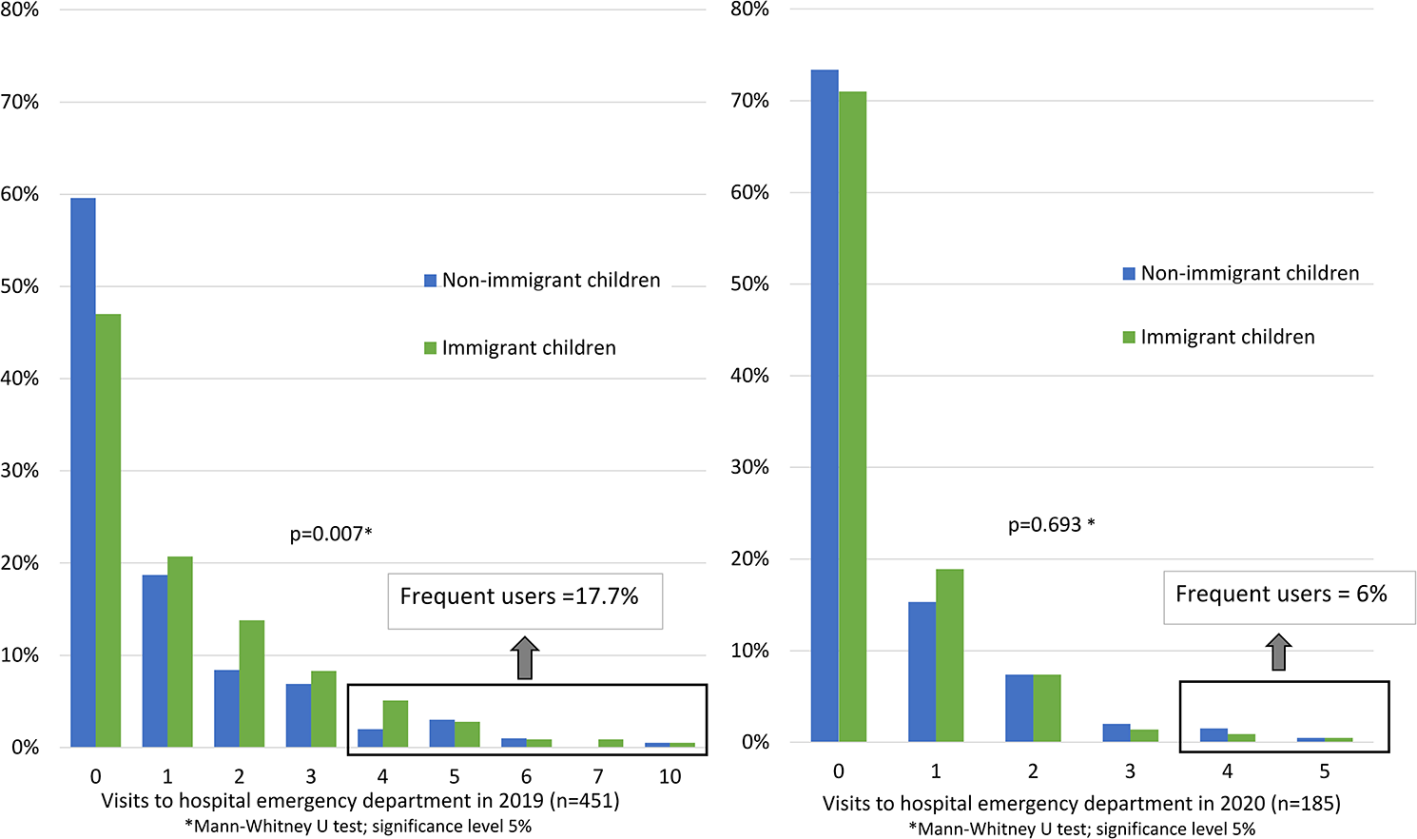
Number of ED visits for immigrant and non-immigrant children in 2019 and 2020

In 2019, 53.5% of immigrant and 40.6% of non-immigrant children (*p* = 0.01) in the CRIAS cohort visited the ED at least once, with an overall higher number of visits by immigrant children (see Fig. 3). Nearly 1 in 5 children were frequent users (19.0% immigrant and 15.9% non-immigrant children; *p* = 0.572) [40], accounting for 39.5% of all visits.

Self-referrals accounted for 86.3% and 71.1% of visits to the ED by immigrant children and non-immigrant children, respectively. In contrast, referrals from PHC accounted for only approximately 3% of visits to the ED, while referrals from the SNS24 helpline were more frequent among non-immigrant children (10.4% vs. 3%) (supplemental Fig. 2a). Over 80% of visits were classified in MTS as low priority, without differences between the 2 groups of children (supplemental Fig. 2b.) [40]. Upper respiratory tract infections, acute gastroenteritis and otitis media were the most frequent overall diagnoses. Among immigrant children, acute gastroenteritis, suppurative otitis media and skin conditions were more frequent (supplemental Table 2). Inpatient admissions occurred in only 2% of visits in 2019, and it is noteworthy that immigrant children were admitted 2 times more frequently than non-immigrant children that same year [40].

In 2020, during the COVID-19 pandemic, ED utilisation was lower, with a dramatic overall reduction of 59% in the number of total visits (see Fig. 3). A sharper decrease in the number of children who visited the ED at least once was observed among immigrant children (46%) when compared with non-immigrant children (34%) [40]. Frequent ED users were reduced to 6%, and inpatient admissions were reduced to 1%. Most visits continued to be self-referrals and were of low clinical priority in both groups (supplemental Fig. 2a.). However, SNS 24 helpline use registered a 2-fold increase, yet parents of immigrant children maintained limited use of the facility, with 7.4% compared to 21.1% of non-immigrant children (*p* = 0.031).

### Parents’ reported reasons to take their children to the hospital emergency department

**Fig. 4:**
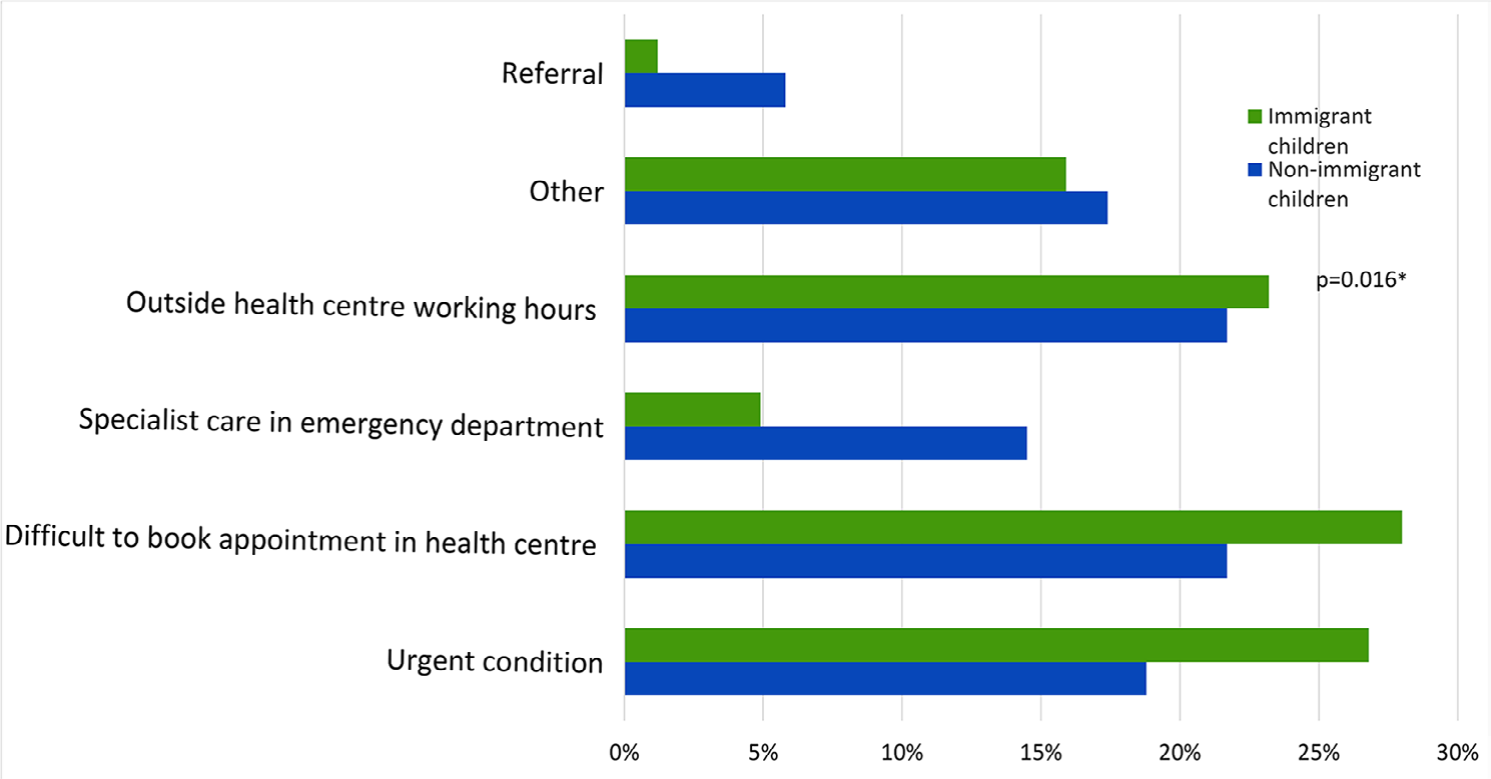
Reasons given by parents for children to visit the emergency department

Parents of 151 children (54.3% immigrant) reported taking their children to the ED in the 3 months prior to enrolment in the study. The main reported reasons to attend are presented in Fig. 4. Difficulties in accessing primary care, including opening hours and perceived urgency of the child’s condition were the most reported reasons to attend ED, particularly among immigrant children [40].

## Discussion

This study analysed differences in healthcare utilisation between immigrant and non-immigrant backgrounds in a paediatric population throughout 2019 and 2020 in the Metropolitan Area of Lisbon. To the best of our knowledge, this is the first study in Portugal comparing immigrant with non-immigrant children and analysing data on public sector PHC and ED utilisation patterns combined with information on the reported use of private practitioners. Some insight is also provided on disruptions in health care utilisation brought about by the COVID-19 pandemic during the transition from 2019 to 2020 in Portugal.

In 2019, no difference in overall PHC utilisation patterns between immigrant and non-immigrant children was documented. This finding is in contrast with a general trend observed in recent research reports where immigrant children tend to use less primary health care and preventive services compared to non-immigrant children [16, 22, 41]. However, among a subset of immigrant children, those born outside Portugal and named “first generation immigrants” in this paper, 36.6% were not given the opportunity to receive the routine child health assessment, which constitutes an integral and important part of PHC preventive services. One possible explanation might be that first-generation immigrant families were not provided with appropriate and timely information about the existence of such assessments and their potential medium- and long-term benefits. This or a more complex combination of factors explaining the drop-out of a major pillar of paediatric health prevention is likely to be the reflection of the general disinvestment in public sector funded Human Resources for Health.

In Amadora, in February 2023, 34% of registered PHC users had no assigned family doctor [42]. In our study, significantly fewer immigrant children had an assigned family doctor, particularly 1st generation immigrant children (see Supplemental Table 1). Children without an assigned regular PHC provider may lack proper continuity of care and opportunities to engage in health promotion, health literacy and disease prevention interventions, thereby likely widening the health inequality gap.

Over the last decade or two, recruitment rounds for medical doctors in the public sector are increasingly left with unfilled places. This may be a result of unsatisfactory working conditions and pay due to years of public sector underfunding and already described in the 2008 WHO Report “Primary Health Care Now More Than Ever“ as disengagement of the state [43]. In Portugal, these circumstances appear to have led general practitioners to leave the Portuguese SNS in recent years, with many moving to the private sector [44]. This situation is now widespread across European countries, where insufficient recruitment and retention of the health workforce in PHC reflect unattractive employment and working conditions together with inadequate strategic planning [45].

 Thirty-two percent of all parents reported not seeking PHC when their child was acutely ill and searching for alternatives. Difficulties in booking and unavailability of appointments at PHC were highlighted, particularly among parents of immigrant children. Noteworthy is the fact that families with additional health insurance coverage outside the SNS or disposing of more financial means tend to have a wider choice between various private care options. This duality of choices based on socioeconomic advantages can perpetuate a “two-tiered” health system and further threatens social justice.

Only 8.4% of non-immigrant families and 2.8% of immigrant families referred using the SNS24 helpline as first contact when their child was ill, illustrating the inadaptation of the SNS24 helpline for paediatric care, particularly for immigrant families, some of whom may face communication difficulties to explain symptoms and signs.

During the COVID-19 pandemic in 2020, the total number of consultations in PHC recorded a reduction of 25%, with immigrant children using services significantly less than non-immigrant children. Of particular concern was the 60% decrease in routine health assessments for all children. A similar trend during the COVID-19 pandemic in the use of PHC by immigrant children in the UK has been reported [17]. Following the first four months of the COVID-19 pandemic in 2020, many doctors in PHC Centres were able to offer some advice by shifting face-to-face consultations to telephone, videocall or email. It is likely that immigrant families may have faced challenges using such options due to a lack of resources, poor digital skills [46], or limited language proficiency.

Close to 50% of all the children in the study used the ED at least once in 2019. One in five children were “frequent users”, accounting for 39.5% of all visits. Nine of the visits to the ED resulted in inpatient admissions, which occurred 2 times more frequently among immigrant children. A higher rate of admission of immigrant children may be related to delay in seeking care or possibly to many of these families having poorer living conditions e.g., overcrowding, not adequate for the treatment of the child. Similar to findings from studies in Europe, including the UK, and the USA, immigrant children had an overall higher utilisation of ED [16, 18]. Difficulties in booking appointments at PHC Centres and possible previous negative experiences in receiving attention or treatment lead parents of immigrant children to seek ED more frequently. In both groups, non-urgent conditions were equally prevalent, contrasting with other research [16] where a larger proportion of immigrant children tend to attend ED for non-urgent conditions.

Acute gastroenteritis, atopic dermatitis and prurigo stropholus were diagnosed more often among immigrant children and highly suggestive of poor living conditions where many immigrant families raise their children. Consistent with other European research [26, 47, 48], immigrant children in the CRIAS study were more frequently living in disadvantaged socioeconomic conditions, compared to children without a migration background. Data from our study suggest that a greater proportion of parents in immigrant families are likely to work in low-skilled jobs and without contracts (supplemental Table 1). In such conditions, leaving work to seek medical care for their child during working hours may not be possible.

An understanding of the factors behind frequent use can support interventions to direct these children to better navigate healthcare and prevention services. A system by which frequent users visiting ED are highlighted to family doctors, who in turn could identify and seek to address possible unmet needs and expectations, has been suggested [49].

Another relevant aspect mentioned in the preference to seek care in the ED relates to the availability of specialist paediatric care and diagnostic tests. Having paediatricians in PHC Centres together with providing resources to perform basic diagnostic tests would in all likelihood reduce pressure on ED services rather than relying on “pseudo-solutions” for the younger paediatric population, such as the SNS 24 phone line.

In 2020, as also found in other studies, ED utilisation reduced dramatically compared with 2019 [50]. This might indicate restrictions to access imposed by the COVID-19 pandemic or probable avoidance by parents to seek the service. Furthermore, it is likely that children spent long periods of time confined at home with less contact with peers, substantially decreasing the risk for acute infections such as upper respiratory tract infections and gastroenteritis, which were the most frequent ED diagnoses in a “typical year” as 2019.

While Thomas Frieden reminds us in The Health Impact Pyramid framework that efforts to address inequalities and the socioeconomic determinants of health have the largest impact on health outcomes [51], quality healthcare systems play a key role in consolidating the base. PHC is best positioned to improve population health and reduce health inequalities but requires adequate financial arrangements [45]. Our study provides evidence suggesting the need for an urgent commitment to strengthen PHC in Portugal. This can be realised through Universal Health Coverage (UHC) reforms by providing adequate resources for the allocation of family doctors to all children, including those recently arrived or with other difficulties, already put forward 15 years ago in the 2008 World Health Report [43]. Additionally, addressing linguistic and cultural barriers through a “diversity competence” approach is critical to facilitate access and interactions between healthcare professionals and healthcare users [52]. Allocating adequate levels of multidisciplinary staffing in PHC Centres could establish PHC at the core of the services network. This may include paediatricians, basic laboratory facilities, speech therapists and enhancing coordination between levels of service such as PHC and ED.

Other fundamental steps include the promotion of intersectoral policies, such as improving labour conditions and social protection, and acknowledging the interconnectedness between health and numerous social determinants. Finally, governance and leadership reforms are necessary to rectify existing challenges [43]. For instance, revising the “two-tiered” health system and the widening health gap between rich and poor created by the commercialisation of health can be critical. An inclusive and participatory governance model should be promoted to address the growing diversity within the population and ensure that the health system remains responsive to the needs of all.

This study presents a number of limitations. Our research focuses on those children using PHC Centers in the SNS, not providing information on those who do not.Those not obtaining residency status are likely to be among non-users of PHC, as they face many more obstacles in accessing public services, including healthcare; another group with absence of information are the children using private health facilities. This limits the representativeness of the study to those who attend public PHC. A further limitation is that data were only available regarding visits to the reference hospital HFF- ED, but it is possible that some children attended other EDs in hospitals outside the Amadora municipality. Additionally, early interruption of recruitment due to the COVID-pandemic resulted in a smaller sample size. A larger sample may have allowed disaggregation of the category ‘non-EU’ countries and could have shed a light on differences in healthcare utilisation across different countries of origin. Some data was collected by questionnaires conducted by interview which could have introduced a social desirability bias. Finally, experiences of accessing and receiving healthcare and non-urgent use of ED by all children require further research on their socio-economic, psychological and cultural dimensions.

## Conclusion

This study identified healthcare utilisation inequalities between immigrant and non-immigrant children. Immigrant children had less access to an assigned family doctor, encountered more frequently barriers in accessing PHC, had more frequent use of ED and faced a dramatic decrease in healthcare utilisation during the COVID-19 pandemic. Both immigrant and non-immigrant children sought ED care mostly for conditions assessed as non-urgent. Furthermore, our results suggest a structural fragility of PHC in Portugal. The slow implementation of planned reforms coupled with political choices in recent decades contributed to a dysfunctional, unequitable and underfunded system largely struggling to meet the diverse needs and expectations of an increasingly varied population. This situation requires national policy and resource allocation strategies to effectively strengthen PHC and intersectoral action, striving to ensure that all children living in Portugal have quality accessible healthcare.

### Electronic supplementary material

Below is the link to the electronic supplementary material.

Supplementary Material 1

## Data Availability

The datasets used in this study are available from the research team coordinator (MROM: mrfom@ihmt.unl.pt) upon reasonable request.
