# Supplementary material for: Patterns of healthcare use among children with immigrant and non-immigrant backgrounds in 2019 and 2020: evidence from the CRIAS cohort study in the metropolitan area of Lisbon, Portugal
Source: BMC Public Health. 2023 Dec 18;23:2531. doi: 10.1186/s12889-023-17402-z (PMC10726586; doi:10.1186/s12889-023-17402-z)
Supplement: Supplementary file 1 — Supplementary Material 1 [file 12889_2023_17402_MOESM1_ESM.docx]

**SUPPLEMENTAL MATERIAL**

**Supplemental table 1. Background characteristics of children and families**

|  | Immigrant children  n (%) | Non-immigrant children  n (%) | Total  n (%) | p value* |
| --- | --- | --- | --- | --- |
|  |  |  |  |  |
|  | 217 (51.7) | 203 (48.3) | 420 (100) |  |
| **Sex of child, n=420** |  |  |  | 0.689 |
| Female | 109 (50.2) | 98 (48.3) | 207 (49.3) |  |
| **Family structure, n=419** |  |  |  | 0.084 |
| Both parents | 99 (45.8) | 117 (57.6) | 216 (51.6) |  |
| Both parents and others | 30 (13.9) | 27 (13.3) | 57 (13.6) |  |
| Single-parent families | 42 (19.4) | 28 (13.8) | 70 (16.7) |  |
| One parent and others | 45 (20.9) | 31 (15.3) | 76 (18.1) |  |
| **Mother educational level**†**, n=384** |  |  |  | 0.059 |
| Lower education | 36 (18.8) | 24 (12.5) | 60 (15.6) |  |
| 9 years completed | 36 (18.8) | 42 (21.9) | 78 (20.3) |  |
| Secondary education | 85 (44.3) | 73 (38.0) | 158 (41.1) |  |
| University degree | 35 (18.2) | 53 (27.6) | 88 (22.9) |  |
| **Occupation**‡, n=414 |  |  |  | <0.001 |
| Low-skilled | 75 (35.2) | 20 (10.0) | 95 (22.9) |  |
| Medium skilled | 99 (46.5) | 102 (50.7) | 201 (48.6) |  |
| High skilled | 34 (16.0) | 69 (34.3) | 103 (24.9) |  |
| Non-defined | 5(2.3) | 10 (5.0) | 15 (3.6) |  |
| **Employment status, n=417** |  |  |  | 0.009 |
| Employed with a contract | 135 (62.5) | 157 (78.1) | 292 (70.0) |  |
| Employed without a contract | 20 (9.3) | 5 (2.5) | 25 (6.0) |  |
| Unemployed | 31 (14.3) | 21 (10.5) | 52 (12.5) |  |
| Self-employed | 16 (7.4) | 9 (4.5) | 25 (6.0) |  |
| Others | 14 (6.5) | 9 (4.5) | 23 (5.5) |  |
| **Household monthly income , n=395** |  |  |  | <0.001 |
| <500 € | 39 (18.5) | 12 (6.7) | 51 (12.9) |  |
| >500—750 | 66 (32.7) | 44(23.3) | 110 (27.8) |  |
| >750—1000€ | 38 (18.5) | 34 (17.6) | 72 (18.2) |  |
| >1000—1500€ | 36 (18.0) | 43 (22.3) | 79 (20.1) |  |
| >1500 | 25 (12.2) | 58 (30.1) | 83 (21.0) |  |
| **Assigned family doctor, n=420** | 161 (73.9) § | 179 (88.6) | 340 (81.0) | <0.001 |
| **Health insurance beyond SNS, n=417** | 63 (29.3) | 104 (51.5) | 167 (40.0) | <0.001 |

Significance level 5%. *Pearson χ2 statistical test

§ 31.7% of 1^st^ generation immigrant children have an assigned doctor

† Based on the International Standard Classification Education

‡Classified as per the Portuguese Classification of professions and summarised in four skill levels according to the International Standard Classification of Occupations

**Supplemental Figure 1. Routine health assessments at age 4 (2019/20) and age 5 (2020/21)**

**Supplemental table 2.** **Most frequent diagnostic categories in ED admissions in 2019**

| **Visits to ED in 2019, n=451** | **Immigrant** **children**  **n (%)** | **Non-immigrant** **children**  **n (%)** | **Total** | **Most frequent diagnosis**  **Per ICD-9-CM chapter**  **n (%)** |
| --- | --- | --- | --- | --- |
|  | 269 (59.6) | 182 (40.4) |  |  |
| **ICD-9-CM, n=440** | 262 (59.5) | 178 (40.5) |  |  |
| **Infectious diseases** | 41 (15.6) | 24 (13.5) | 65 (14.8) | *Acute gastroenteritis 37 (56.9) |
| **Nervous system and sense organs** | 31 (11.8) | 14 (7.9) | 45 (10.2) | **Otitis media 35 (77.8) |
| **Respiratory diseases** | 74 (28.2) | 47 (26.4) | 121 (27.5) | Upper respiratory tract infections 84 (69.4) |
| **Digestive diseases** | 8 (3.1) | 17 (9.6) | 25 (5.7) | Constipation 18 (72.0) |
| **Skin and subcutaneous tissue diseases** | 26 (9.9) | 11 (6.2) | 37 (8.4) | ***Prurigo strophulus 18 (48.6)  Atopic dermatitis 8 (21.7) |
| **Symptoms, signs and ill- defined conditions** | 51 (19.5) | 48 (27.0) | 99 (22.5) | Fever 30 (30.3) |
| **Injury and poisoning** | 26 (9.9) | 10 (5.6) | 36 (8.2) | Traumatic lesions 17 (47.2) |
| **Others** | 5 (1.9) | 7 (3.9) | 12 (2.8) | Urinary tract infection 4  Hip synovitis 2 |
| * Gastroenteritis more frequent among immigrant children; p=0.005 | | | | |
| ** Supurative otitis media more frequent among immigrant children; p=0.032 | | | | |
| *** Strophulus and atopic dermatitis more frequent among immigrant children; p=0.053 | | | | |

**Supplemental figure 2a. Origin of child on arrival at ED in 2019 and 2020**

**Supplemental figure 2b. Manchester Triage Classification 2019 and 2020**
